# Evolution of socioeconomic inequalities in oral health and use of dental services in adult population of Brazil between 2013 and 2019

**DOI:** 10.1590/0102-311XEN162324

**Published:** 2025-09-01

**Authors:** Ana Karine Macedo Teixeira, Paulo Roberto Borges de Souza

**Affiliations:** 1 Faculdade de Farmácia, Odontologia e Enfermagem, Universidade Federal do Ceará, Fortaleza, Brasil.; 2 Instituto de Comunicação e Informação Científica e Tecnológica em Saúde, Fundação Oswaldo Cruz, Rio de Janeiro, Brasil.

**Keywords:** Health Inequalities, Oral Health, Health Inequality Indicators, Desigualdades de Saúde, Saúde Bucal, Indicadores de Desigualdade em Saúde, Inequidades en Salud, Salud Bucal, Indicadores de Desigualdad en Salud

## Abstract

The Brazilian National Oral Health Policy has increased access to dental services for the Brazilian population. However, it is not clear whether there has been a reduction in oral health inequalities in the country. The aim of the study was to investigate the evolution of socioeconomic inequalities in oral health, the use of oral hygiene products, and the use of dental services in the adult population of Brazil between 2013 and 2019. Data from the *Brazilian National Health Survey* conducted in 2013 (n = 60,202) and 2019 (n = 88,531) were used to calculate the slope index of inequality (SII) and relative index of inequality (RII) in terms of schooling and family income per capita. The dependent variables were the use of a toothbrush, toothpaste, and dental floss, functional dentition, use of dental services at least once in life, use of dental services in the previous year, and use of dental services for preventive care. Inequalities in the use of oral hygiene products and the use of dental services reduced between 2013 to 2019. However, functional dentition maintained the same levels of inequality in terms of schooling (RII = 1.6) and income (RII = 1.3). Schooling inequalities in the use of preventive dental care increased (SII = 33.3 in 2013, SII = 38.9 in 2019). This study underscores the need to reorient the Brazilian National Oral Health Policy in order to reduce tooth loss and improve the use of dental services for preventive care in the most vulnerable groups. Despite improvements in the use of dental services and oral hygiene products, socioeconomic inequalities in oral health persist in Brazil.

## Abstract (pt)

A Política Nacional de Saúde Bucal no Brasil ampliou o acesso aos serviços odontológicos para a população brasileira, mas não está claro se houve redução das desigualdades em saúde bucal no país. Este estudo avaliou a evolução das desigualdades socioeconômicas em saúde bucal, uso de produtos de higiene bucal e utilização de serviços odontológicos pela população adulta entre 2013 e 2019. Dados da *Pesquisa Nacional de Saúde* realizada em 2013 (n = 60.202) e 2019 (n = 88.531) foram utilizados para calcular o índice de desigualdade absoluta (SII, acrônimo em inglês) e o índice relativo de desigualdade (IRD) por escolaridade e renda familiar *per capita*. As variáveis dependentes foram o uso de escovas dentais, dentifrício e fio dental, dentição funcional, uso de serviços odontológicos pelo menos uma vez na vida, uso de serviços odontológicos no último ano e uso de serviços odontológicos para cuidados preventivos. As desigualdades no uso de produtos de higiene bucal e no uso de serviços odontológicos diminuíram entre 2013 e 2019. No entanto, a dentição funcional manteve os mesmos níveis de desigualdade em termos de escolaridade (IRD = 1,6) e renda (IRD = 1,3). Por sua vez, as desigualdades escolares no uso de cuidados odontológicos preventivos aumentaram (SII = 33,3 em 2013, SII = 38,9 em 2019). Este estudo destaca a necessidade de reorientar a Política Nacional de Saúde Bucal para reduzir a perda dentária e melhorar a utilização dos serviços odontológicos para cuidados preventivos nos grupos mais vulneráveis. Apesar das melhorias no uso de serviços odontológicos e no acesso a produtos de higiene bucal, persistem desigualdades socioeconômicas em saúde bucal no Brasil.

## Abstract (es)

La Política Nacional de Salud Bucal de Brasil ha incrementado el acceso a los servicios odontológicos para la población brasileña, pero no está claro si se ha producido una reducción de las desigualdades en materia de salud bucal en el país. El objetivo del estudio fue evaluar la evolución de las desigualdades socioeconómicas en materia de salud bucal, el uso de productos de higiene oral y el uso de los servicios odontológicos en la población adulta de Brasil entre 2013 y 2019. Se utilizaron datos de la *Encuesta Nacional de Salud de Brasil* realizada en 2013 (n = 60.202) y 2019 (n = 88.531) para calcular el índice de desigualdad absoluta (SII, por su sigla en inglés) y el índice relativo de desigualdad (IRD) según nivel educativo e ingresos familiares per cápita. Las variables dependientes incluyeron el uso de cepillo de dientes, pasta dental e hilo dental, dentición funcional, uso de servicios dentales al menos una vez en la vida, uso de servicios dentales en el último año y uso de servicios dentales para cuidados preventivos. Las desigualdades en el uso de productos de higiene bucal y en el uso de servicios dentales se redujeron entre 2013 y 2019. Sin embargo, la dentición funcional mantuvo los mismos niveles de desigualdad en términos de educación (IRD = 1,6) e ingresos (IRD = 1,3). Mientras tanto, las desigualdades escolares en el uso de la atención dental preventiva aumentaron (SII = 33,3 en 2013, SII = 38,9 en 2019). Este estudio destaca la necesidad de reorientar la Política Nacional de Salud Bucal de Brasil con el fin de reducir la pérdida de dientes y mejorar el uso de los servicios dentales para la atención preventiva en los grupos más vulnerables. Pese las mejoras en el uso de los servicios dentales y el acceso a los productos de higiene y servicios dentales, persisten las desigualdades socioeconómicas en salud bucal en Brasil.

## Introduction

Oral health conditions are a major public health problem, affecting around 3.5 billion people throughout the world in 2019, and are considered the health problems that most affect humanity ^1^ ^,^ ^2^. However, oral problems do not affect the population in a uniform manner. The burden of oral diseases has more than doubled in low-income countries over the past thirty years, which constitutes a challenge for healthcare systems ^2^.

In Brazil, the implementation of the Brazilian National Oral Health Policy (“Smiling Brazil”) in 2004 constituted a major turning point in the country’s oral healthcare system. This policy reoriented the practices of oral health teams towards a health vigilance model, including oral health promotion and prevention actions, and expanded access to dental services in both primary health care and specialized care ^3^ ^,^ ^4^. As a result, improvements have occurred in the country’s oral health indicators ^5^ ^,^ ^6^ ^,^ ^7^.

However, improving oral health in Brazil in recent years has been marked by regional, socioeconomic, racial, and cultural inequalities, as well as difficulties in gaining access to oral health care, which implies a higher burden of oral diseases among the most vulnerable groups ^7^ ^,^ ^8^ ^,^ ^9^ ^,^ ^10^ ^,^ ^11^. The monitoring of health inequalities generates data to assist in the establishment of equity-oriented policies, programs, and practices ^12^ ^,^ ^13^. However, it is unclear whether inequalities in oral health have diminished since the implementation of the Brazilian National Oral Health Policy.

Monitoring income and educational inequality is fundamental. Lifelong learning and a minimum income for a healthy life have been recommended for addressing determinants of health and health equity on the global agenda ^12^ in order to leave no one behind in the process of economic, social, and environmental development ^13^. Corassa et al. ^7^ found improvements in oral health indicators, such as toothbrush/toothpaste/floss use and a reduction in edentulism between 2013 and 2019 based on data from the *Brazilian National Health Survey* (PNS, acronym in Portuguese), which is the same database used in the present study. However, the authors did not investigate whether a decrease in oral health inequalities occurred among different income and schooling strata in the period. This makes the present study relevant, as it assesses whether the improvement in oral health indicators was accompanied by a reduction in socioeconomic inequalities.

Other studies have used complex measures to analyze oral health inequalities ^10^ ^,^ ^14^ ^,^ ^15^ ^,^ ^16^ ^,^ ^17^. However, few have conducted this analysis over time, as done in the present investigation ^11^ ^,^ ^18^ ^,^ ^19^. Complex health inequality measures are important and consider information from all social strata. Such measures are sensitive to the distribution of the population among socioeconomic groups ^13^ ^,^ ^20^.

Monitoring oral health inequalities over time enables assessing the impact of oral health policies on the most vulnerable groups, as it implies greater health gains among groups with worse social conditions. Thus, the aim of this study was to investigate the evolution of socioeconomic inequalities in oral health, the use of oral hygiene products, and the use of dental services in the adult population of Brazil between 2013 and 2019.

## Methods

This study used data from the PNS conducted in 2013 and 2019, which is a nationwide household-based survey with probabilistic sampling. The survey targets residents in both urban and rural areas ^21^.

Participants were selected using a 3-stage cluster sampling process. Census tracts or tract groups were initially selected as the primary sampling units. Households were then selected from the National Register of Addresses for Statistical Purposes [CNEFE - Cadastro Nacional de Endereços para Fins Estatísticos]. Lastly, one resident per household was selected to participate in individual interviews. The PNS is part of the Integrated System of Household Surveys [SIPD - Sistema Integrado de Pesquisas Domiciliares] of the Brazilian Institute of Geography and Statistics (IBGE, acronym in Portuguese) and the primary units were selected from the Master Sample, maintaining its stratification ^22^.

For this study, we included individuals 18 years of age or older, resulting in a sample of 60,202 interviewees in 2013 and 88,531 in 2019. The two editions of the PNS are representative of the Brazilian population. Despite the fact that the sample was larger in 2019, which increased the precision of the estimates, this did not affect comparability between the two periods ^23^. Furthermore, the same questionnaire for the oral health module was used in both editions.

The PNS 2013 received approval from the Brazilian National Research Ethics Committee in June 2013 (protocol n. 328,159) and the PNS 2019 received approval in August 2019 (protocol n. 3,529,376). The PNS database is publicly available at https://www.ibge.gov.br/estatisticas/sociais/saude/9160-pesquisa-nacional-de-saude.html?=&t=microdados.

Five outcomes were considered in this study. These outcomes are indicators recommended by the World Health Organization (WHO) ^24^ and therefore relevant for monitoring inequalities in oral health. The variables were selected from the oral health module of the PNS:

(1) Use of a toothbrush, toothpaste, and dental floss to clean teeth (yes/no): self-reported use;

(2) Functional dentition (yes/no): defined as the presence of 20 or more teeth in the mouth ^24^ based on the self-report of the number of missing teeth in the upper and lower arches;

(3) Use of dental services at least once in life (yes/no): self-reported;

(4) Use of dental services in the previous year (yes/no): self-reported;

(5) Use of dental services for prevention in the previous visit (yes/no): defined as the most recent visit to a dentist for the purposes of cleaning, prevention, or revision among those who had at least one dental visit in the previous year. In 2013, the answer to this question was “cleaning, revision, maintenance, or prevention”. In 2019, the answer to the same question was “cleaning, prevention, or revision”. This change in wording did not affect the meaning of the answer and did not impact the comparability of this indicator between the two editions of the PNS.

Socioeconomic status of the participants was investigated based on family income per capita and schooling level. Family income per capita in minimum wages (sum of all income received, including pensions and other benefits, divided by the number of residents in the household) was categorized in quintiles: up to 1, > 1-2, > 2-3, > 3-5, and > 5. Schooling (identified by the highest level of education achieved) was classified into six groups (Brazilian classification): no schooling, incomplete primary school education, complete primary school education, incomplete high school education, complete high school education, and higher education (incomplete or higher).

The sample was stratified by sex, age in years (18-29, 30-59, 60 or older), household income per capita, and schooling for 2013 and 2019. A chi-square test was then performed to determine the prevalence of oral health variables and the use of dental services by year.

An equiplot was created to show the distribution of oral health indicators and the use of dental services according to socioeconomic strata for each year analyzed. While inequality indices (relative index of inequality - RII and slope index of inequality - SII) measure absolute and relative differences between income and schooling distribution groups, the equiplot shows the prevalence of the indicator in each income and schooling category for the two editions of the PNS, thus enabling the comparison of estimates between 2013 and 2019 as well as between the categories of socioeconomic variables for each period. This serves to complement the information obtained with the inequality indices.

Inequalities in oral health and dental service use were measured using the SII and RII. These indices employ regression measures that consider the entire socioeconomic distribution rather than comparing only two extremes ^13^ ^,^ ^20^.

A Ridit score was assigned to each income and schooling category based on the midpoint of the cumulative distribution of participants in a given category. Individuals were cumulatively ranked from 0 to 1 according to their ascending socioeconomic position ^20^.

The SII is the absolute difference in the prevalence of outcome variables and the RII is the prevalence rate ratio between the uppermost stratum group and lowermost group. An SII value greater than 0 and an RII value greater than 1 indicates that individuals with higher socioeconomic status are more likely to have a better oral health status and greater use of dental services. The model was adjusted for age and sex, as these variables were related to oral health conditions and the use of dental services.

The SII and RII were estimated using Poisson regression models, with oral health indicators as the dependent variable and the Ridit score related to socioeconomic status, sex, age, and year of the survey as independent variables. A 2-way interaction term between the inequality indices and year of the survey was included in the models but only retained in the final model if the coefficient of the interaction term was significantly different from 0. SII was estimated for each year as the difference between the prevalence predicted by the model with a Ridit score of 1 and the prevalence predicted by the model with a Ridit score of 0. Similarly, the RII was calculated as the ratio of the predicted prevalence rates at the two extremes of the Ridit score.

To analyze the data, the databases of the two editions of the PNS were appended and the year of the survey was linked to the stratification variables. The analysis was carried out in the Stata 13.0 software (https://www.stata.com), using the survey module to account for the sample design and sampling weight to obtain the estimates.

## Results

Women and individuals between 30 and 59 years of age constituted the majority of the sample in both 2013 and 2019. A slight decrease occurred in the proportion of adults with an incomplete primary school education or no schooling. Family income per capita remained constant between both periods, with most individuals belonging to the social stratum earning up to the monthly minimum wage (Table 1).

**Table 1:** Distribution of adult population according to sociodemographic variables. *Brazilian National Health Survey*, 2013 and 2019.

Variables	2013		2019	
	%	95%CI	%	95%CI
Sex				
Female	52.9	52.1-53.7	53.2	52.6-53.8
Male	47.1	46.3-47.9	46.8	46.2-47.4
Age group (years)				
18-29	26.1	25.5-26.8	22.1	21.5-22.7
30-59	55.8	55.1-56.5	56.3	55.6-57.0
60 or +	18.1	17.5-18.6	21.6	21.1-22.2
Schooling				
No formal education	8.3	7.9-8.7	6.1	5.8-6.4
Incomplete primary school	30.7	29.9-31.4	28.7	28.1-29.3
Complete primary school	9.9	9.5-10.4	7.8	7.4-8.1
Incomplete high school	5.6	5.2-5.9	6.7	6.4-7.0
Complete high school	28.1	27.4-28.8	29.8	29.2-30.4
Higher education (incomplete or higher)	17.4	16.6-18.2	20.9	20.2-21.7
Family income per capita (minimum wage)				
Up to 1	49.7	48.8-50.7	51.2	50.4-52.0
> 1-2	28.8	28.1-29.5	28.2	27.6-28.8
> 2-3	9.4	8.9-9.8	9.1	8.7-9.4
> 3-5	6.4	6.0-6.8	6.3	6.0-6.7
> 5	5.7	5.2-6.2	5.2	4.8-5.6

95%CI: 95% confidence interval.

Regarding oral health variables, the use of a toothbrush, toothpaste, and dental floss improved from 52.8% in 2013 to 62.9% in 2019 (p < 0.0001) and the proportion of individuals with functional dentition increased from 76.9% to 78.7% (p < 0.0001) (Table 2). In terms of the use of dental services, an increase was found in the proportion of individuals who visited a dentist at least once in life and in the previous last year. However, the proportion of individuals who used dental services for prevention at their last visit decreased from 53.1% in 2013 to 47.6% in 2019 (p < 0.0001) (Table 2).

**Table 2:** Prevalence of oral health variables and use of dental services according to year of the *Brazilian National Health Survey* (2013 and 2019).

Variables	2013		2019	
	%	95%CI	%	95%CI
Use of toothbrush, toothpaste, and dental floss	52.8	51.8-53.7	62.9	62.2-63.7 *
Functional dentition (20 or more teeth)	76.9	76.3-77.6	78.7	78.2-79.1 *
Use of dental services at least once in life	96.7	96.4-96.9	98.2	98.0-98.3 *
Use of dental services in previous year	44.3	43.5-45.1	49.1	48.5-49.8 *
Use of dental service for prevention at last visit	53.1	51.2-54.4	47.6	46.7-48.5 *

95%CI: 95% confidence interval.

* p < 0.0001 (chi-square test).

**Figure 1:**
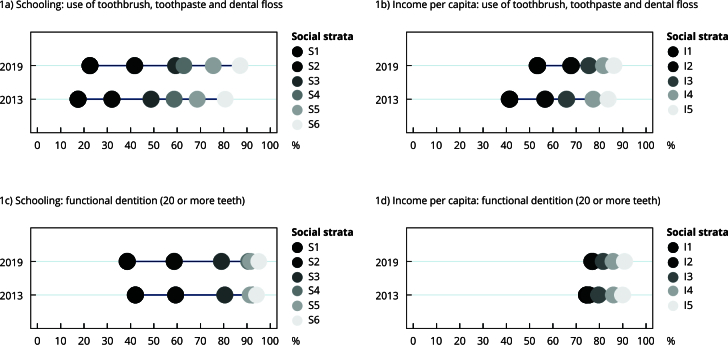
Prevalence of use of toothbrush, toothpaste, and dental floss and functional dentition according to social strata of income and schooling. *Brazilian National Health Survey*, 2013 and 2019.

Note: Social strata - Schooling (S1: no schooling, S2: incomplete primary school education, S3: complete primary school education, S4: incomplete high school education, S5: complete high school education, S6: higher education, incomplete or higher); Income per capita in minimum wages (I1: up to 1, I2: > 1-2, I3: > 2-3, I4: > 3-5, I5: > 5).

**Figure 2:**
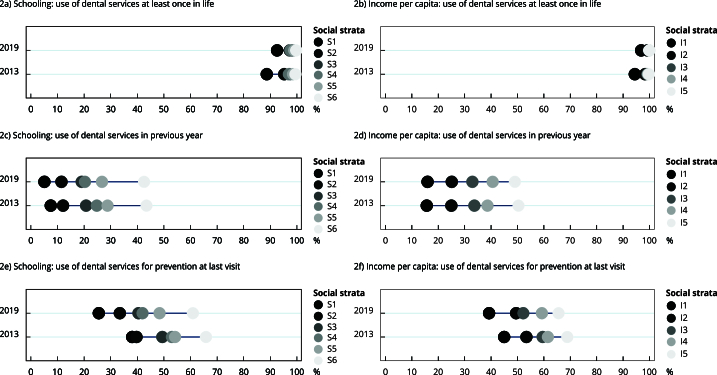
Prevalence of use of dental services at least once in life, use of dental services in previous year, and use of dental services for prevention according to social strata of income and schooling. *Brazilian National Health Survey*, 2013 and 2019.

Note: Social strata - Schooling (S1: no schooling, S2: incomplete primary school education, S3: complete primary school education, S4: incomplete high school education, S5: complete high school education, S6: higher education, incomplete or higher); Income per capita in minimum wages (I1: up to 1, I2: > 1-2, I3: > 2-3, I4: > 3-5, I5: > 5).

The distribution of outcomes by schooling and family income per capita are shown in the equiplots (Figures 1 and 2). Individuals with a higher socioeconomic status had better oral health and dental service use indicators, with a similar linear pattern found in both 2013 and 2019. Comparing the two periods, the most vulnerable strata increased the proportion of toothbrush, toothpaste, and dental floss use in 2019 as well as the use of dental services at least once in life and in the previous year. Moreover, education seems to discriminate inequality in terms of oral health and the use of dental services more strongly than income, particularly with regards to functional dentition, as the group with the lowest schooling level had worse outcomes than the group with the lowest family income per capita.

Absolute and relative inequalities by income and schooling decreased between 2013 and 2019 with regards to the use of a toothbrush, toothpaste, and floss and the use of dental services at least once in life. The RII for the use of dental services in the previous year by schooling also decreased from 3.0 (95%CI: 2.8-3.1) in 2013 to 2.6 (95%CI: 2.5-2.7) in 2019 and use by income decreased from 2.6 (95%CI: 2.5-2.8) in 2013 to 2.2 (95%CI: 2.1-2.3) (Table 3).

Despite having improved in the country, the same disparity in functional dentition was found in the two periods. In relative terms, the presence of 20 or more teeth remained 1.6 (95% confidence interval - 95%CI: 1.5-1.6) times and 1.3 (95%CI: 1.3-1.3) times higher in the better-educated and higher-income groups, respectively. In contrast, an increase in inequality in the use of dental services for preventive care in the previous year was found according to schooling; the RII was 1.9 (95%CI: 1.8-2.1) in 2013 and increased to 2.3 (95%CI: 2.1-2.5) in 2019. No change in inequality was found in the analysis by family income per capita (Table 3).

**Table 3:** Absolute and relative inequality indices according to schooling and income per capita. *Brazilian National Health Survey*, 2013 and 2019.

	SII				RII			
	2013	95%CI	2019	95%CI	2013	95%CI	2019	95%CI
Schooling *								
Use of toothbrush, toothpaste, and dental floss	69.0	66.6-71.4	64.6 **	62.4-66.7	3.7	3.5-3.9	2.7 **	2.6-2.8
Functional dentition (20 or more teeth)	36.5	34.6-38.4	36.9	35.2-38.6	1.6	1.6-1.7	1.6	1.5-1.6
Use of dental services at least once in life	9.6	8.6-10.5	5.6 **	5.1-6.2	1.1	1.1-1.1	1.0 **	1.0-1.1
Use of dental services in previous year	48.1	44.5-50.7	49.5	47.3-51.7	3.0	2.8-3.1	2.6 **	2.5-2.7
Use of the dental service for prevention at last visit	33.3	29.3-37.3	38.9 **	35.6-42.1	1.9	1.8-2.1	2.3 **	2.1-2.5
Income per capita *								
Use of toothbrush, toothpaste, and dental floss	52.2	49.7-54.7	46.1 **	44.2-48.0	2.7	2.5-2.8	2.0 **	1.9-2.0
Functional dentition (20 or more teeth)	20.5	18.6-22.4	22,3	20.8-23.8	1.3	1.3-1.3	1.3	1.3-1.3
Use of dental services at least once in life	8.3	7.4-9.2	4.5 **	4.1-5.0	1.1	1.1-1.1	1.0 **	1.0-1.0
Use of dental services in previous year	43.4	40.8-45.8	41.1	39.0-43.1	2.6	2.5-2.8	2.2 **	2.1-2.3
Use of dental service for prevention at last visit	29.6	25.9-33.4	30.0	27.2-32.8	1.8	1.6-1.9	1.9	1.8-2.0

95%CI: 95% confidence interval; RII: relative index of inequality; SII: slope index of inequality.

* Adjusted for sex and age;

** p < 0.05.

## Discussion

This study demonstrated a decrease in inequalities in the use of oral hygiene products and dental services over time. The results showed an increase in the use of oral hygiene products among the most vulnerable groups and that the policy of expanding oral health services in the country has managed to reach adults with lower income and schooling.

Despite these improvements, notable inequalities persist in Brazil. Although the most vulnerable groups increased their use of dental services, this was not sufficient to narrow the gap in tooth loss compared to those with a higher socioeconomic status. Moreover, a reduction in the use of preventive dental services occurred in the most vulnerable group, which led to an increase in inequality with regards to this indicator.

In contrast to our findings, other studies ^11^ ^,^ ^18^ that assessed oral health inequality over time found an increase in inequality. However, such studies used different methods, outcomes, age groups, and periods for the analysis. While we investigated the adult population 18 years of age and older who self-reported their oral health status, the studies cited analyzed adolescents ^11^ and older people ^18^ submitted to oral examinations. Furthermore, these studies investigated the period from 2003 to 2010, which was the beginning of the implementation of the Brazilian National Oral Health Policy ^3^, while the present study considered the period of expansion of the policy from 2013 to 2019, which may explain the difference in the results. Similar to our findings, another study reported a reduction in inequality in the use of dental services in Brazil, although it did not assess inequality by schooling ^19^.

Poverty and vulnerability in Brazil decreased from 2008-2009 to 2017-2018 ^25^, possibly reducing inequality in the use of oral hygiene products over time. Although these are expensive items ^8^ ^,^ ^26^, this study showed that the most vulnerable group increasingly uses toothbrushes, toothpaste, and dental floss to maintain oral hygiene, likely due to the implementation of the Brazilian National Oral Health Policy, the aim of which is to ensure access to oral hygiene items for the most vulnerable population ^3^.

One study showed that schooling and flossing were the factors that best explained the difference in the average number of missing teeth between income strata ^17^. Although our study indicated improvements in schooling and flossing in the adult Brazilian population, such improvements did not reduce inequalities in functional dentition. This is likely due to the fact that tooth loss is the result of chronic diseases, such as dental caries and periodontal disease, with cumulative effects throughout life that require more time for changes in epidemiological patterns to emerge. A period of six years (2013-2019) may be too short to observe changes in the inequality of tooth loss, although it is possible to see an improvement in the population average over the same period.

In this study, the impact of schooling on inequality with regards to functional dentition was more pronounced, as shown in the equiplot graph. This phenomenon may be attributed to the higher level of health literacy in individuals with a higher level of schooling. Consequently, such individuals have better self-care practices, better communication with healthcare providers, a deeper understanding of prevention measures, the optimized use of health resources, and healthier dietary habits ^27^. Such habits encompass the appropriate use of oral hygiene instruments and low sugar intake, both of which exert an impact the occurrence of tooth loss.

Aspects related to the use of dental services, income, self-care habits, and dental pain have been identified as determinants of tooth loss ^28^. Limited access to specialized dental services for conservative treatment, whether in the public or private sector, and the ease with which tooth extraction is permitted in situations of pain reveal a injuring model of care that remains embedded in the country’s oral health policy. This model forces vulnerable populations to undergo numerous tooth losses rather than obtaining conservative treatment ^28^ ^,^ ^29^. Even when primary health units in Brazil have an adequate physical infrastructure, such units offer a high number of tooth extraction procedures instead of preventing tooth loss ^30^.

The reduction in inequality in the use of dental services throughout life and in the previous year, as opposed to the increase in inequality in the use of services for preventive purposes, suggests that the most socioeconomically disadvantaged groups have been increasingly able to access dental services over the years. However, this was achieved using a curative healthcare model, likely reinforced by the poorer oral health status in the most vulnerable groups requiring dental treatment.

Other studies have also indicated the unequal use of dental services for preventive care ^10^ ^,^ ^31^. Low use of preventive dental care services leads to the delayed detection of diseases, reducing the likelihood of the implementation of conservative treatments and, consequently, increasing the risk of tooth loss. An inverse correlation has been found between the availability of dental services and oral health needs. Oral health providers are often overconcentrated in wealthier areas, where disease rates are generally lower ^2^. Consequently, access to preventive dental care is challenging for the most vulnerable population.

Moreover, political instability, budget restrictions on health policies, including oral health, the loss of the federal direction of the Brazilian National Oral Health Policy, and the flexibilization of the Brazilian National Primary Care Policy revised in 2017 ^32^ ^,^ ^33^ ^,^ ^34^ ^,^ ^35^ may be reflected in the increased inequality in the use of dental services for preventive purposes.

Despite the progress made by the Brazilian National Oral Health Policy up to 2013, there has been a decrease in the number of oral health teams in the population coverage indicator since 2016 and a 41.5% decrease in the coverage of oral health promotion and prevention actions between 2015 and 2017 ^32^. However, exclusive dental health insurance expanded in the same period in Brazil ^34^.

Despite the improvement in oral health indicators and the reduction in disparities in the use of dental health services and oral hygiene products demonstrated in this study, inequalities in oral health by income and schooling persist throughout the country. Although most oral diseases can be prevented through the implementation of self-care measures, such measures are contingent upon broader population-level interventions that address the social, economic, and political determinants of health ^2^.

The increase in the Human Development Index and reduction in the country’s Gini index have contributed significantly to improved oral health ^36^. Higher levels of schooling and income are associated with adopting healthy behaviors, greater access to hygiene products, and greater use of healthcare services ^37^ ^,^ ^38^ ^,^ ^39^. It is therefore necessary to reduce inequalities in income and education in the country, as these aspects exert a direct impact on the oral health of the population. It is particularly necessary to reduce inequality in education, as groups with the lowest schooling levels had worse oral health and less access to dental services than groups with the lowest income.

A limitation of the present study is that the functional dentition outcome was identified from the self-report of tooth loss by the participants of the PNS, which could have overestimated or underestimated the number of missing teeth due to memory or information bias, as respondents tend to answer according to their perception of health. However, another study showed good agreement between self-reported tooth loss and clinical assessments in surveys ^40^. Another limitation is that we did not investigate the frequency of tooth brushing/day. Thus, self-reported use of a toothbrush, toothpaste, and dental floss may not necessarily indicate a good frequency and adequate control of dental biofilm. Future studies should assess inequalities considering other determinants of oral health and the use of dental services, such as race, place of residence, and region of the country, which were not covered in this study.

The global sustainable development agenda reiterates the call for equity, with the aim of ensuring a healthy life and promoting well-being for all. To this end, monitoring health inequalities is a priority, provided it is based on disaggregated data that enable tracking progress in health goals among disadvantaged subgroups ^13^. This study contributes to the monitoring of the oral health status of vulnerable groups. Groups with lower income and schooling showed an improvement in the use of dental services and oral hygiene products in the period investigated, pointing to equity in the policy of dental service use in the country. However, no increase in equity occurred with regards to functional dentition and the use of dental services for prevention.

Lastly, the Brazilian National Oral Health Policy must be oriented to reduce inequalities and implement strategies to prevent tooth loss in vulnerable groups. Access to oral hygiene products, including dental floss, should also be increased and access to preventive dental services should be expanded in groups with lower schooling and income.
